# Publisher Correction to: BMC Rheumatology, volume 3

**DOI:** 10.1186/s41927-019-0083-6

**Published:** 2019-08-20

**Authors:** BMC Rheumatology

**Affiliations:** London, UK

**Publisher Correction to: BMC Rheumatology, volume 3**

An error occurred during the publication of a number of articles in *BMC Rheumatology*. Several articles were published in volume 3 with a duplicate citation number.

In this correction article the old and new citation metadata are published in Table 1.

**Table 1:** Overview of incorrect and correct citation metadata

DOI	Incorrect citation number	Correct citation number
10.1186/s41927-019-0067-6	1	23
10.1186/s41927-019-0069-4	4	20
10.1186/s41927-019-0070-y	2	24
10.1186/s41927-019-0071-x	3	22
10.1186/s41927-019-0072-9	5	21

The original articles have been updated. The publisher apologizes for the inconvenience caused to our authors and readers.

